# Supplementary material for: All-in-one, bio-inspired, and low-power crypto engines for near-sensor security based on two-dimensional memtransistors
Source: Nat Commun. 2022 Jun 23;13:3587. doi: 10.1038/s41467-022-31148-z (PMC9226122; doi:10.1038/s41467-022-31148-z)
Supplement: Supplementary file 1 — Supplementary Information [file 41467_2022_31148_MOESM1_ESM.docx]

Supplementary Information

All-in-one, bio-inspired, and low-power crypto engines for near-sensor security based on two-dimensional memtransistors

Akhil Dodda,^1^ Nicholas Trainor^2^, Joan. M . Redwing,^2,3^ and Saptarshi Das^1,2,34,*^

*^1^ Engineering Science and Mechanics, Penn State University, University Park, PA 16802, USA*

*^2^Materials Science and Engineering, Penn State University, University Park, PA 16802, USA*

*^3^Materials Research Institute, Penn State University, University Park, PA 16802, USA*

*^4^Electrical Engineering and Computer Science, Penn State University, University Park, PA 16802, USA*

***Supplementary Table 1***

| *Supplementary Table 1: Benchmarking Emerging IoT Platforms* | | | | | |
| --- | --- | --- | --- | --- | --- |
| *Material* | | *Sensing* | *Storage* | *Security* | *Reference* |
| 2D | MoS_2_ | Y | N | N | ^14^ |
|  | MoS_2_, WS_2_, MoSe_2_, WSe_2_ | N | Y *(Electronic)* | N | ^15^ |
|  | WSe_2_/MoS_2_/h-BN/HfS_2_ /WSe_2_/MoS_2_ | N | Y *(Electronic)* | N | ^16^ |
|  | Graphene/MoS_2-x_ O_x_/Graphene | N | Y *(Electronic)* | N | ^17^ |
|  | Graphene/MoS_2_ | N | Y *(Electronic)* | N | ^18^ |
|  | Graphene/h-BN/MoS_2_ | N | Y *(Electronic)* | N | ^19^ |
|  | Graphene/h-BN/MoS2 | N | Y *(Electronic)* | N | ^20^ |
|  | Graphene/MoS_2_ | Y | Y *(Optical)* | N | ^21^ |
|  | h-BN/WSe_2_-  h-BN/WSe_2_ | Y | Y *(Optical)* | N | ^22^ |
|  | MoS_2_/PTCDA | Y | Y *(Optical)* | N | ^23^ |
|  | MoS_2_/Au-Nano Particles | Y | Y *(Optical)* | N | ^24^ |
|  | WSe_2_/h-BN | Y | Y *(Optical)* | N | ^25^ |
|  | MoS_2_/PbS | Y | Y *(Optical)* | N | ^26^ |
|  | BP/Al_2_O_3_ | N | Y *(Electronic)* | N | ^27^ |
|  | BP/h-BN/MoS_2_ | N | Y *(Electronic)* | N | ^28^ |
|  | BP/Al_2_O_3_  /BP/Al_2_O_3_ | N | Y *(Electronic)* | N | ^29^ |
|  | MoS_2_/Metal Nano Crystal | N | Y *(Electronic)* | N | ^30^ |
|  | MoS_2_ | Y | N | N | ^31^ |
|  | MoS_2_ | Y | Y *(Electronic)* | N | ^32^ |
|  | MoS_2_/PZT | Y | Y *(Electronic)* | N | ^33^ |
|  | WSe_2_ | Y | N | N | ^34^ |
|  | MoO_x_/MoS_2_ | N | Y *(Electronic)* | N | ^35^ |
|  | MoS_2_ | N | N | Y | ^36^ |
| Oxide Based  Memristors | Ag:SiO_2_ or MgO/HfO_2_:Ag | N | Y *(Electronic)* | N | ^37^ |
|  | TiN/TaO*_x_*  /HfAl*_y_*O*_x_*/TiN | N | Y *(Electronic)* | N | ^38^ |
|  | ITO/LaAlO3  /SrTiO3 | N | Y *(Electronic)* | N | ^39^ |
|  | Ag_2_S | N | Y *(Electronic)* | N | ^40^ |
|  | Indium Gallium Zinc Oxide (IGZO) | N | Y *(Electronic)* | Y | ^41^ |
|  | Al_2_O_3_/TiO_2–x_ | N | Y *(Electronic)* | Y | ^42^ |
|  | Ag:SiO_2_ | N | Y *(Electronic)* | Y | ^43^ |
|  | Ag:SiO_2_ | N | Y *(Electronic)* | Y | ^44^ |
|  | TiO_2_ | N | Y *(Electronic)* | Y | ^45^ |
| Phase Change  Materials | GeSbTe(GST) | Y | N | N | ^46^ |
| Nano Crystals (NC) | B-doped Si NC | Y | Y *(Optical)* | N | ^47^ |
| ***2D*** | ***This Work*** | ***Y*** | ***Y (Electronic)*** | ***Y*** |  |

***Supplementary Figure 1***

***Supplementary Figure 1.*** *Optical image of the all-in-one bio-inspired crypto chip.*


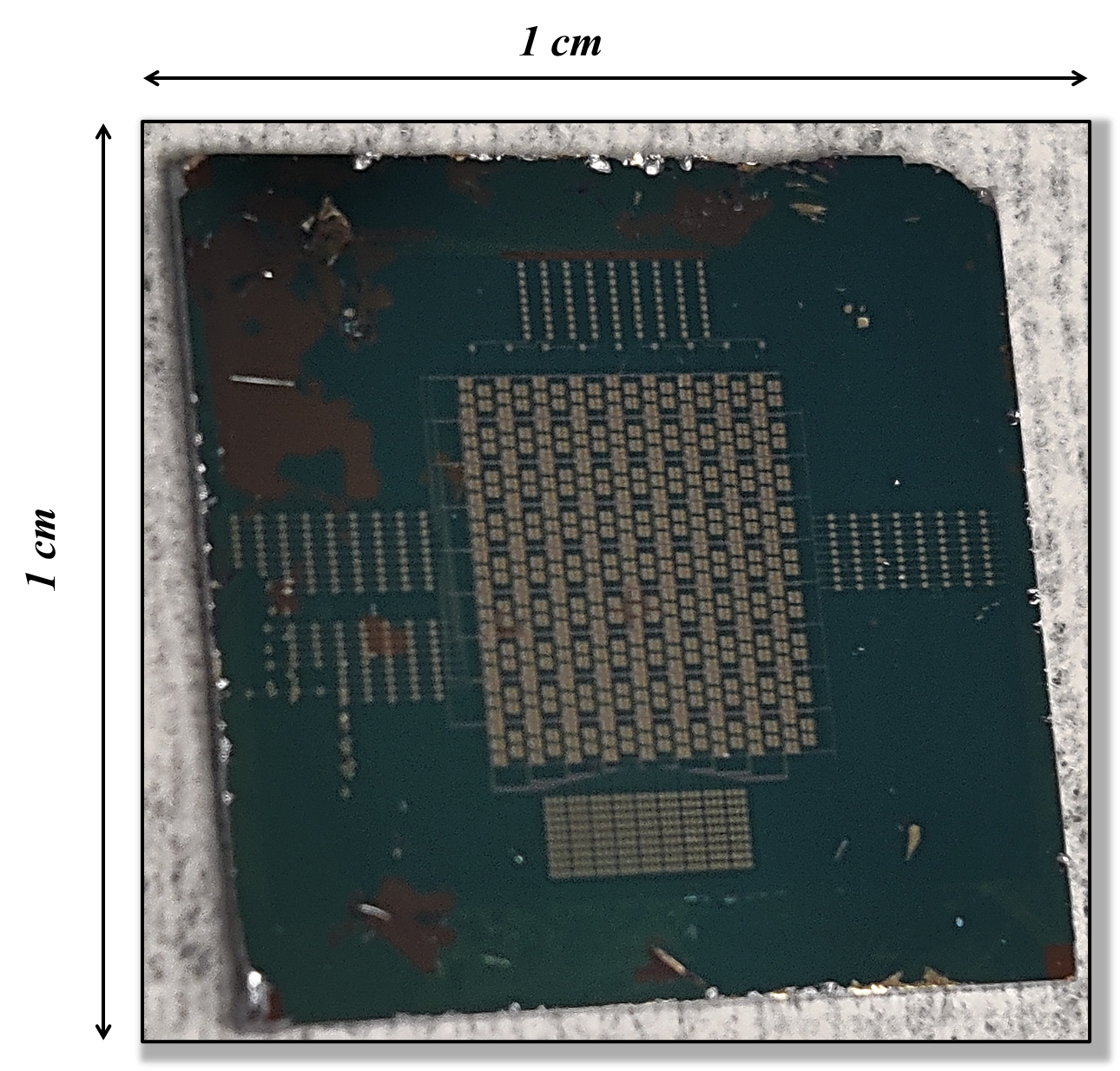


***Supplementary Figure 2***

***Supplementary Figure 2.*** *Optical image of the crossbar array of the crypto engines. Note that several (20) optical images were taken at 5X resolution using a Nikon LV150M microscope and stitched together in photoshop.*


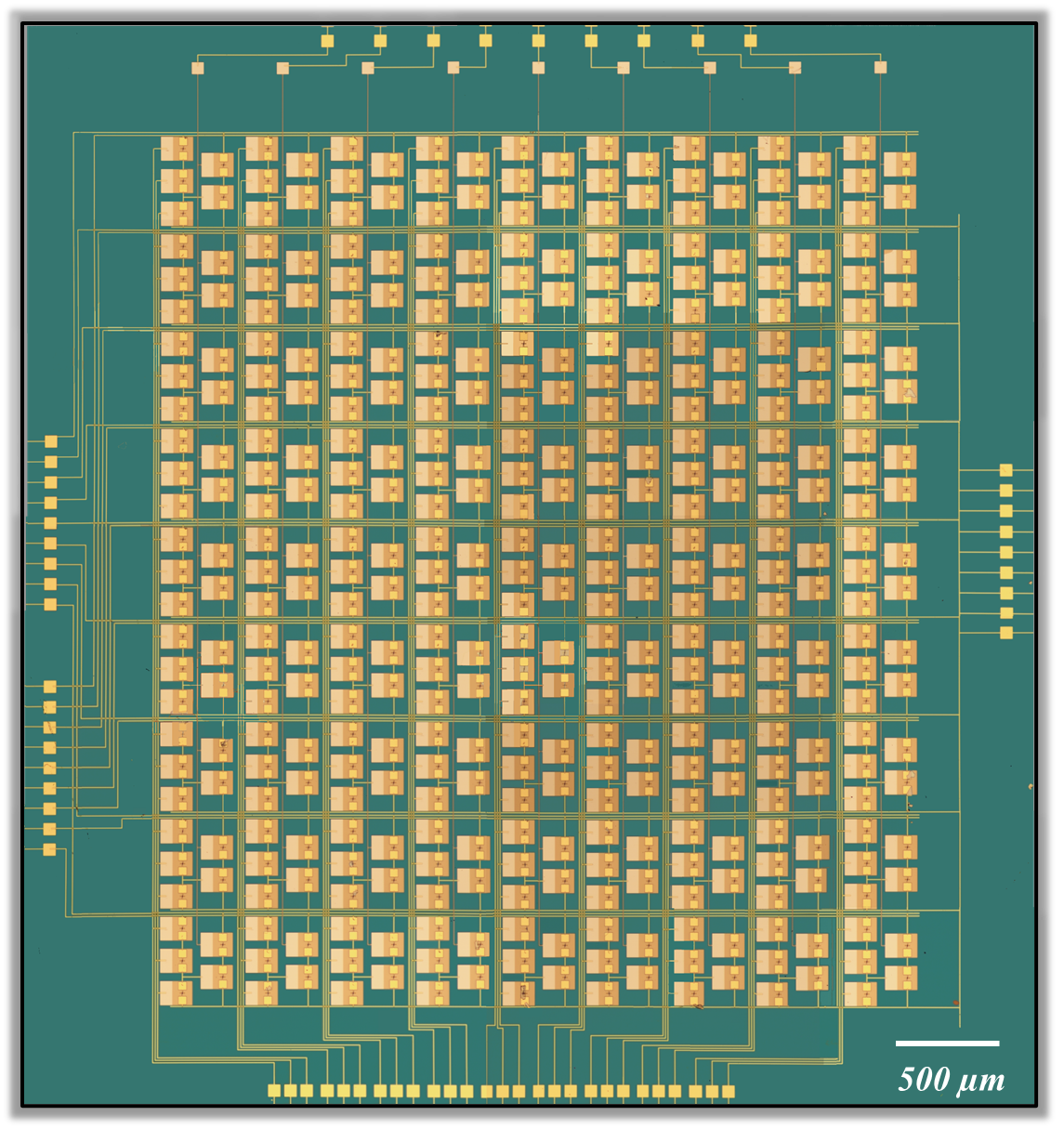


***Supplementary Figure 3***

***Supplementary Figure 3.*** *Optical image individual MoS_2_ memtransistors, which are locally back-gated using a stack comprising of 50 nm Al_2_O_3_ on 50/20 nm Pt/TiN. All back-gate islands were placed on SiO_2_/p^++^-Si substrate. The Al_2_O_3_/Pt/TiN gate islands allow non-volatile programming of MoS_2_ memtransistors as well as enhance the photoresponse of MoS_2_ memtransistors owing to the phenomenon of gate-tunable persistent photoconductivity, thereby empowering our hardware platform to enable in-memory computing and near-sensor security.*


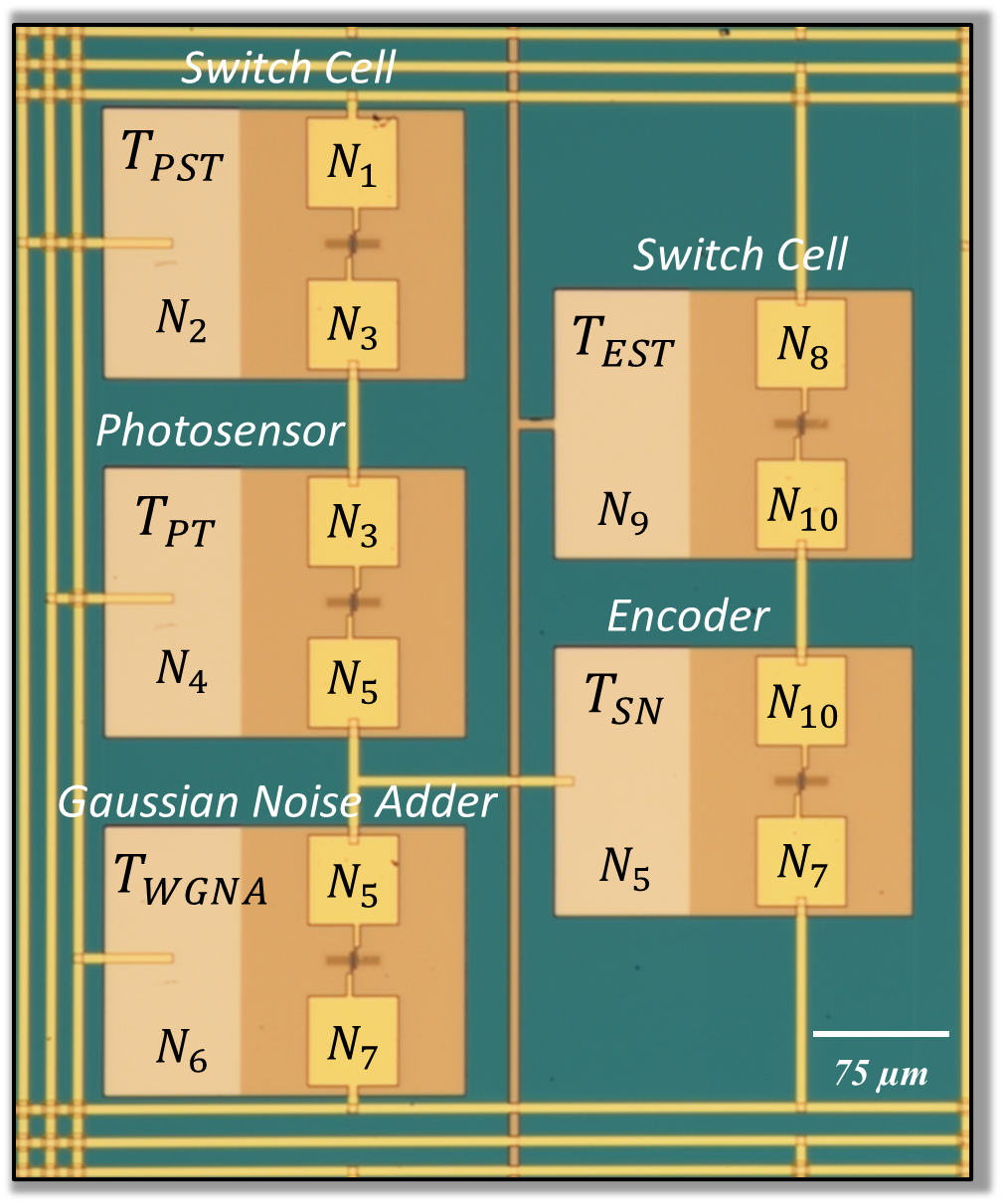


***Supplementary Figure 4***

***Supplementary Figure 4.*** *Circuit schematic of the crossbar architecture of the crypto engines. The nodes* $N_{2}$*,* $N_{4}$*,* $N_{6}$*, and* $N_{9}$ *from the crypto engines in a given column are connected to common* $V_{N2}$*,* $V_{N4}$*,* $V_{N6}$ *and* $V_{N9}$ *lines, respectively, and nodes* $N_{1}$*,* $N_{7}$*, and* $N_{8}$ *from the crypto engines in a given row are connected to common* $V_{N1}$*,* $V_{N7}$*, and* $V_{N8}$ *lines, respectively. This allows us to select any crypto engine corresponding to a given row and column*.


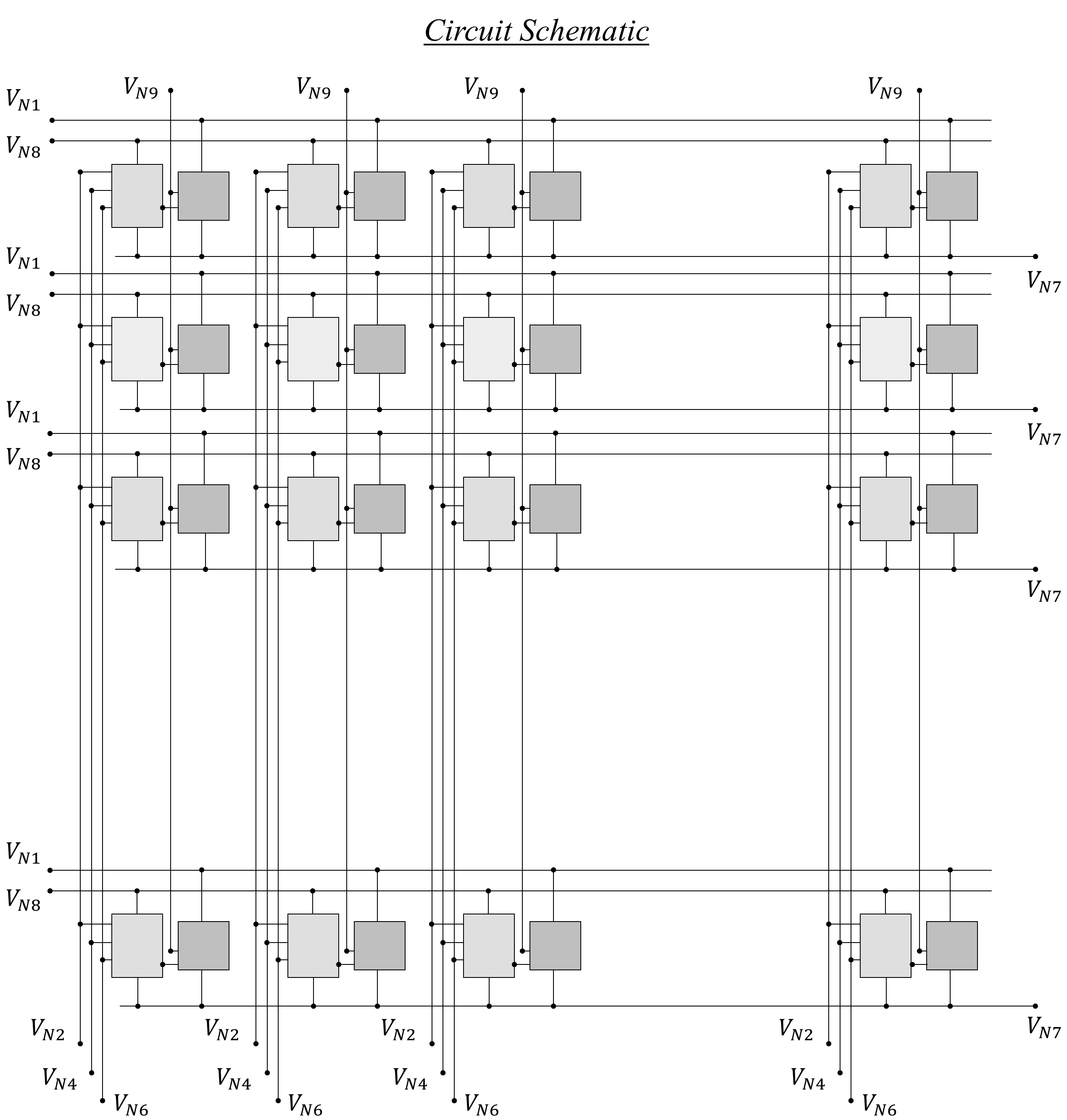


***Supplementary Figure 5***

***Supplementary Figure 5.*** *The Circuit schematic of an individual crypto engine****.*** *The memtransistors* $T_{\mathrm{PST}}$ *and* $T_{\mathrm{PT}}$ *are connected in series at the node,* $N_{3}$*,* $T_{\mathrm{PT}}$ *and* $T_{\mathrm{WGNA}}$ *are connected in series at the node,* $N_{5}$*, which is also connected to the local back-gate of* $T_{\mathrm{SN}}$*, and finally,* $T_{\mathrm{EST}}$ *and* $T_{\mathrm{SN}}$ *are connected in series at the node,* $N_{10}$*. The nodes,* $N_{2}$*,* $N_{4}$*,* $N_{6}$*, and* $N_{9}$*, respectively, serve as the local back-gate terminals of* $T_{\mathrm{PST}}$*,* $T_{\mathrm{PT}}$*,* $T_{\mathrm{WGNA}}$*, and* $T_{\mathrm{EST}}$*, node* $N_{1}$ *serves as the drain terminal of* $T_{\mathrm{PST}}$*, node* $N_{8}$ *serves as the drain terminal of* $T_{\mathrm{EST}}$*, and node* $N_{7}$*serves as the common source terminal for* $T_{\mathrm{WGNA}}$ *and* $T_{\mathrm{SN}}$*.*


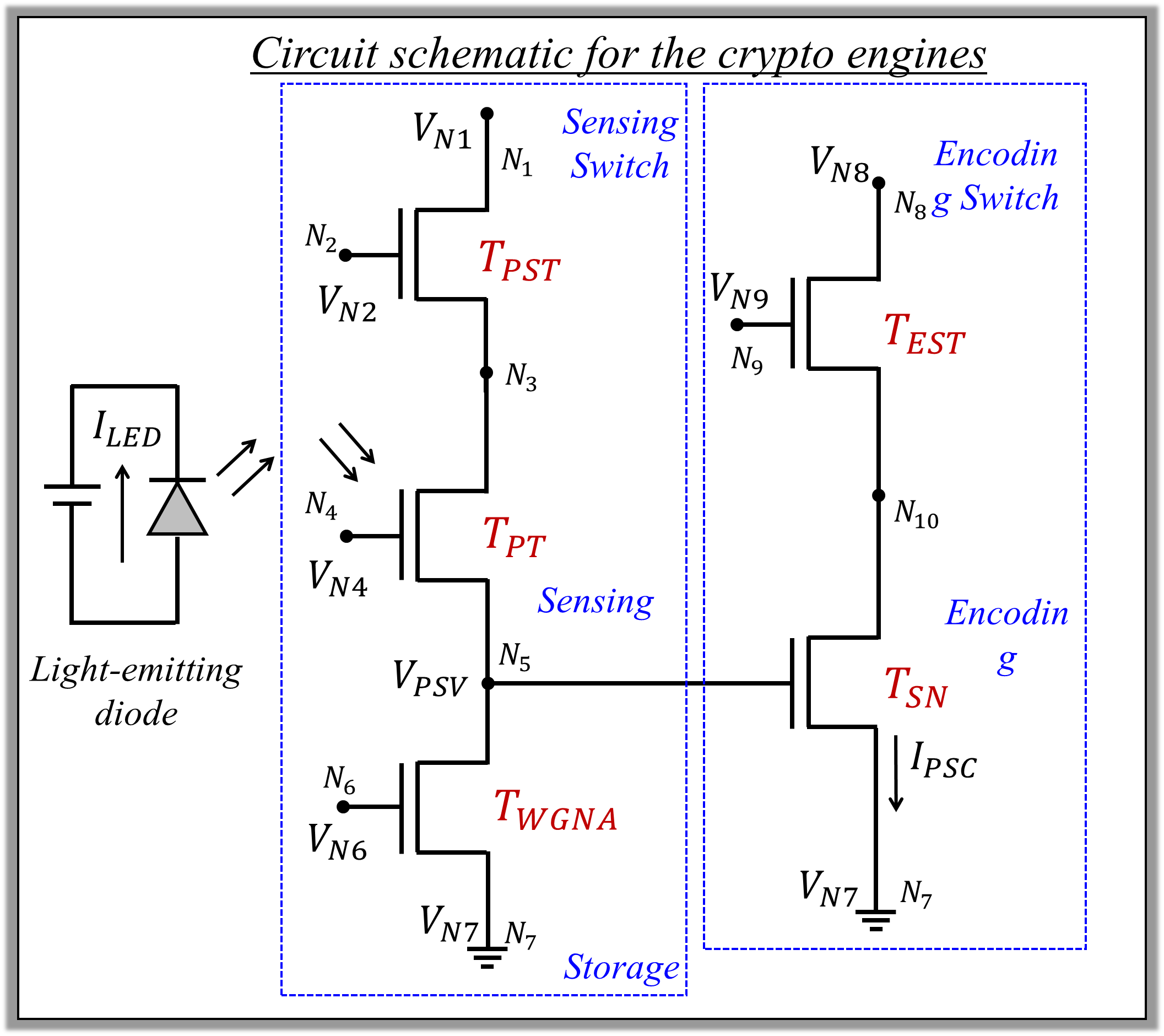


***Supplementary Figure 6***

***Supplementary Figure 6****. a) Transfer characteristics i.e., source to drain current (*$I_{\mathrm{DS}}$*) as a function of the local back-gate voltage (*$V_{\mathrm{BG}}$*) for 64 monolayer MoS_2_ memtransistors across the 8 × 8 array of the crypto engines measured at source to drain voltage,* $V_{\mathrm{DS}}$ *= 1 V. The channel length (*$L$*) and channel width (*$W$*) of each MoS_2_ memtransistors are 1 µm and 5 µm, respectively.* *Colormaps of distribution of b) current on/off ratio (*$r_{ON/OFF}$*), c) threshold voltage (*$V_{\mathrm{TH}}$*) extracted at iso-current of 100 nA/µm, and d) subthreshold slope (SS) over 3 orders of magnitude change in* $I_{\mathrm{DS}}$*, of 64 MoS_2_ memtransistors. Extracted mean values for* $r_{ON/OFF}$*,* $V_{\mathrm{TH}}$*, and SS, were found to be, ~10^6^, ~1.65 V and ~355 mV/decade, respectively, with corresponding standard deviation values of ~10^6^, ~0.42 V and ~85 mV/decade respectively.*


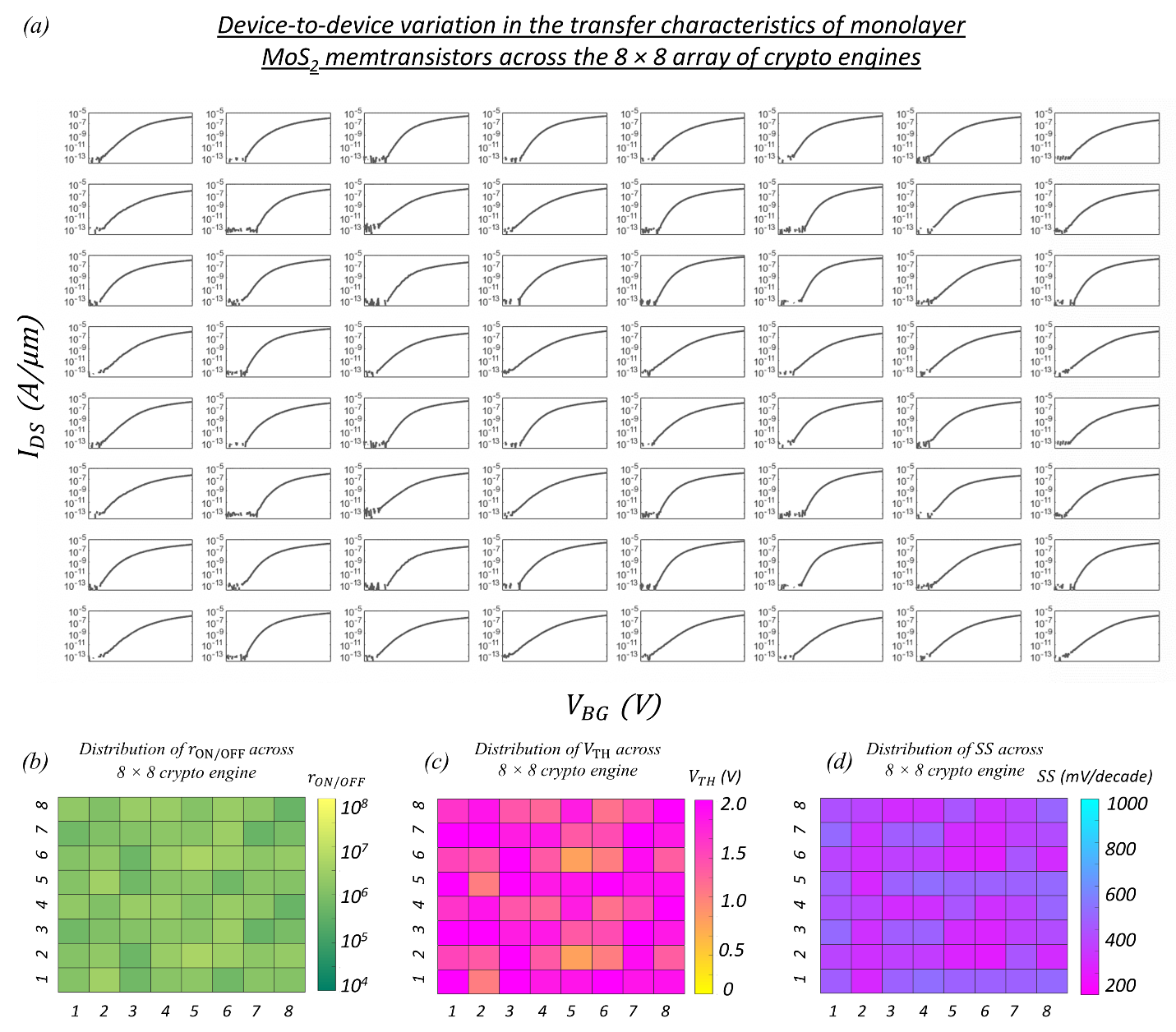


***Supplementary Figure 7***

***Supplementary Figure 7.*** *a) Long-term retention of two representative post-programmed analog conductance states for ~10^4^ seconds. b) Evolution of the memory ratio (*$MR$*) between these two programmed conductance states (brown)) and the corresponding exponential fitting (blue) given by,* $MR$ *=* ${MR}_{0}$*exp(-t/τ), where* ${MR}_{0}$ *= 600, τ = 7.6×10^3^ s. The projected time before the* $MR$ *reaches 1, i.e. these two states become indistinguishable is found* *to be ~14 hours*.


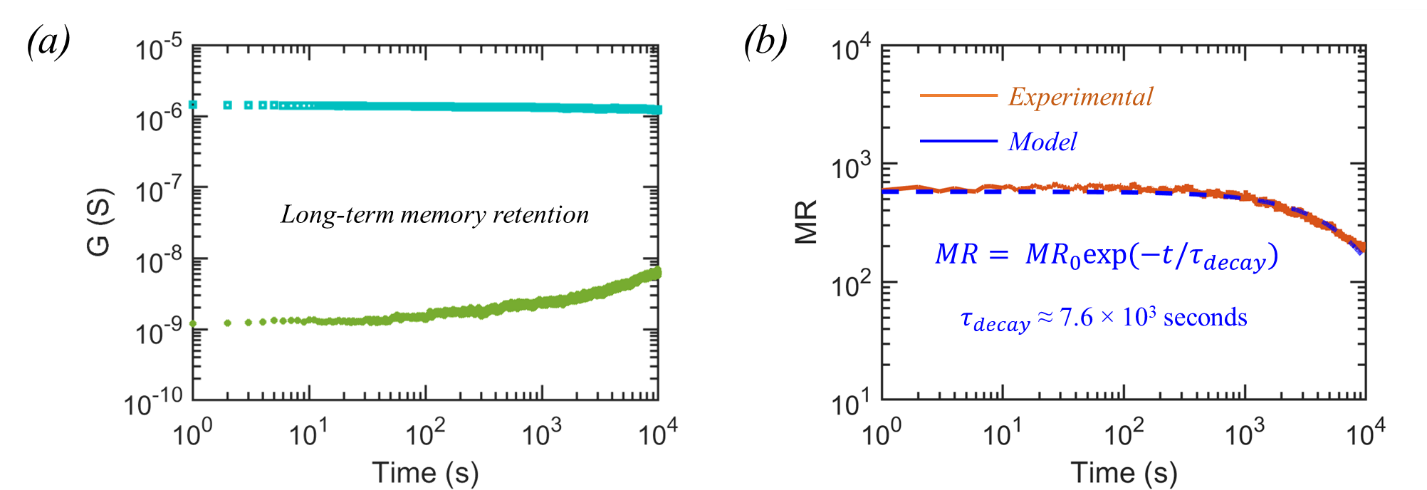


***Supplementary Figure 8***

***Supplementary Figure 8.*** *a) Memory endurance when successively programmed and erased to achieve two representative analog conductance states for 2 × 10^3^ cycles with programming and erase voltage of*$V_{P}$ *= -9 V and* $V_{E}$ *= 10 V respectively applied for duration of 100 ms.b) Evolution of memory ratio (*$MR$*) as a function of memory cycle (green) and the corresponding power law fitting (pink) given by,* $MR$ *=* ${MR}_{0}t^{\gamma}$*, with* $\gamma$ *~ -0.1. The projected memory endurance before the* $MR$ *reaches 1 is found to be 5 × 10^8^ cycles.*


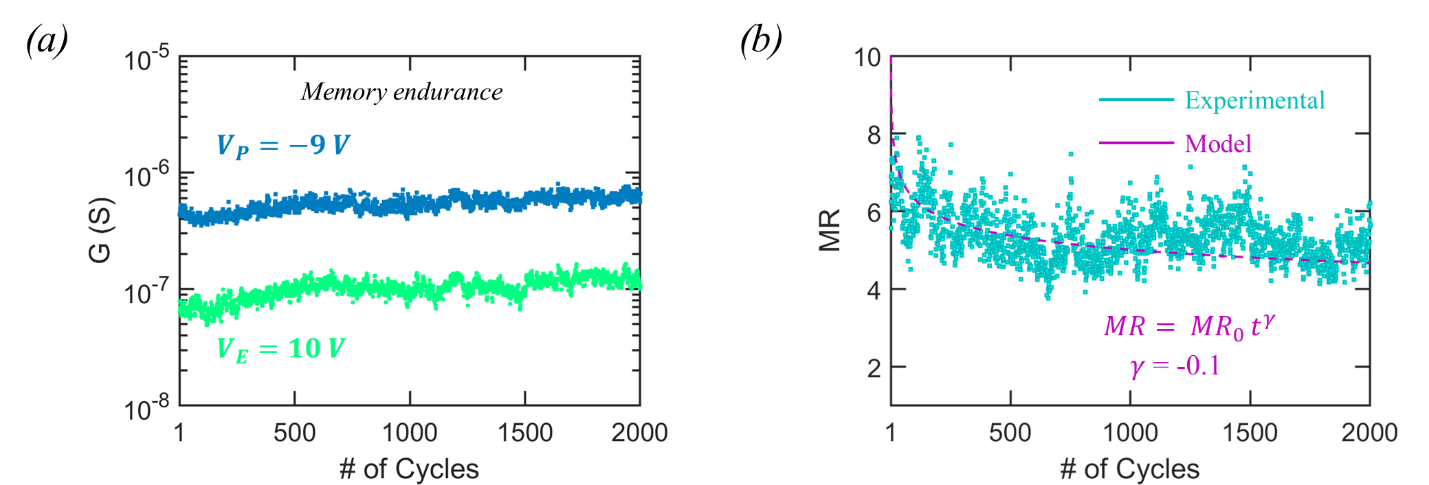


***Supplementary Figure 9***

***Supplementary Figure 9.*** *a) Transfer characteristics of 64 monolayer MoS_2_ memtransistors across the 8 × 8 array of the crypto engines measured before (blue) and after programming (pink) with* $V_{P}$*= -10 V for 1 second. b) Compiled device-to-device variation in programmability. c) Colormap of distribution of memory ratio (*$MR$*) measured at* $V_{\mathrm{BG}}$ *= 0 V for these 64 MoS_2_ memtransistors. The mean and standard deviation values for* $MR$ *were found to be 2.8×10^5^ and standard deviations of 8.7×10^5^, respectively.*


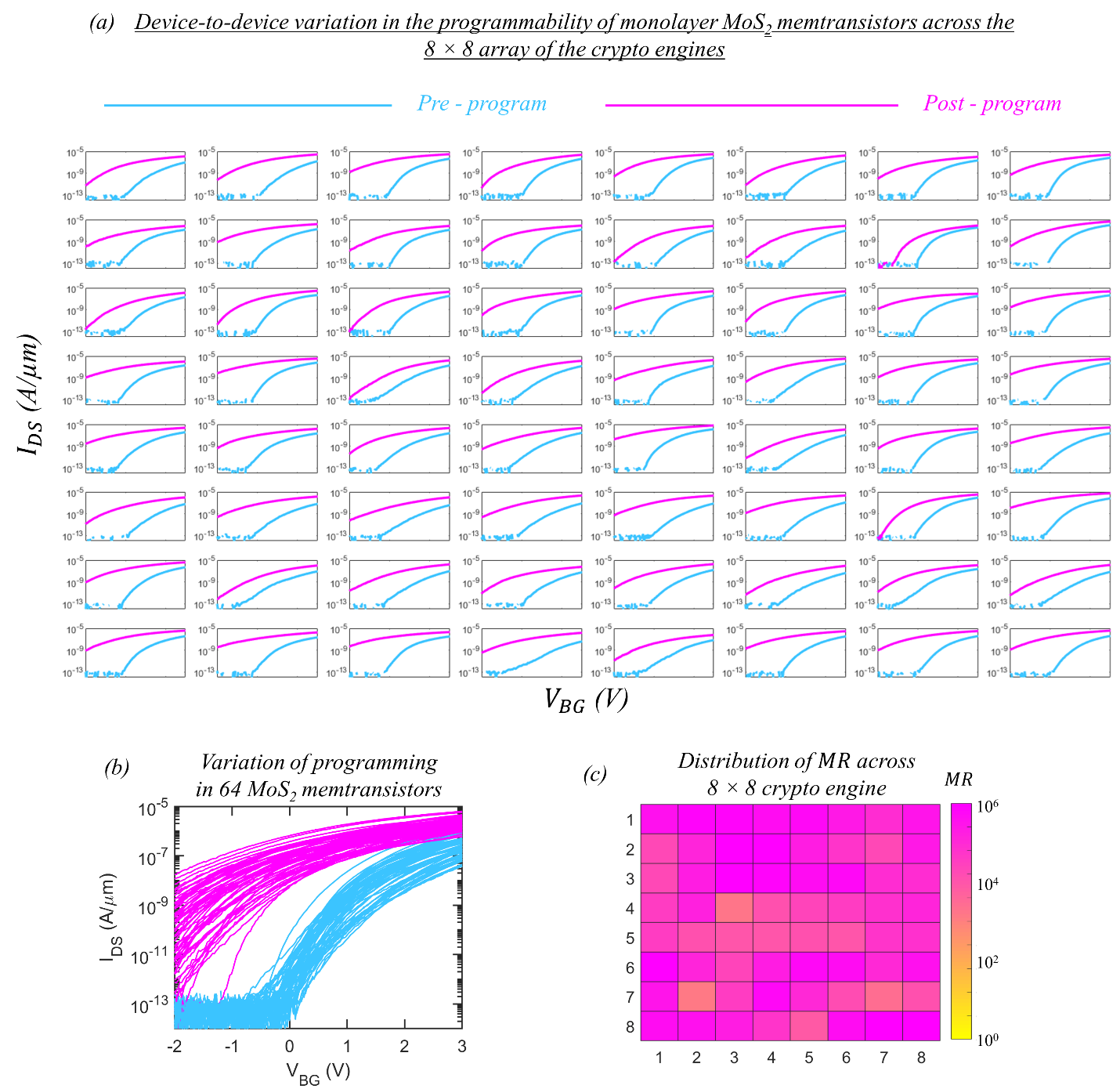


***Supplementary Figure 10***

***Supplementary Figure 10.*** *a) Programming and b) erase energy expenditures, calculated based on* $E_{P/E}=\frac{1}{2}C_{G}{V_{P/E}}^{2}$*, where* $C_{G}$ *is the capacitance for the local back-gate.*


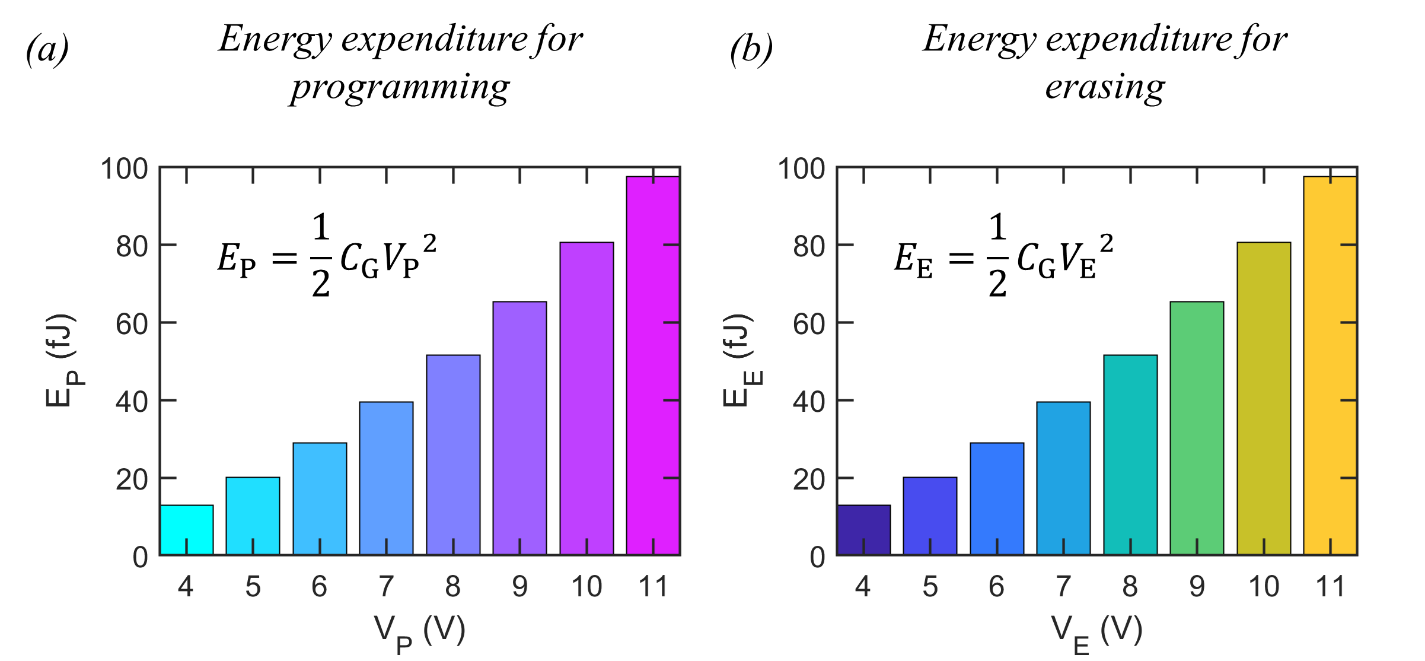


***Supplementary Figure 11***

***Supplementary Figure 11.*** *a) Transfer characteristics of a representative MoS_2_ memtransistor measured after exposure to* $I_{\mathrm{LED}}$ *= 20 mA at different* $V_{\mathrm{illumin}}$ *for different* $t_{\mathrm{illumin}}$*. b)* *Colormap of* $r_{PH}$ *as a function of* $V_{\mathrm{illumin}}$ *and* $t_{\mathrm{illumin}}$ *for* $I_{\mathrm{LED}}$ *= 20 mA****.***


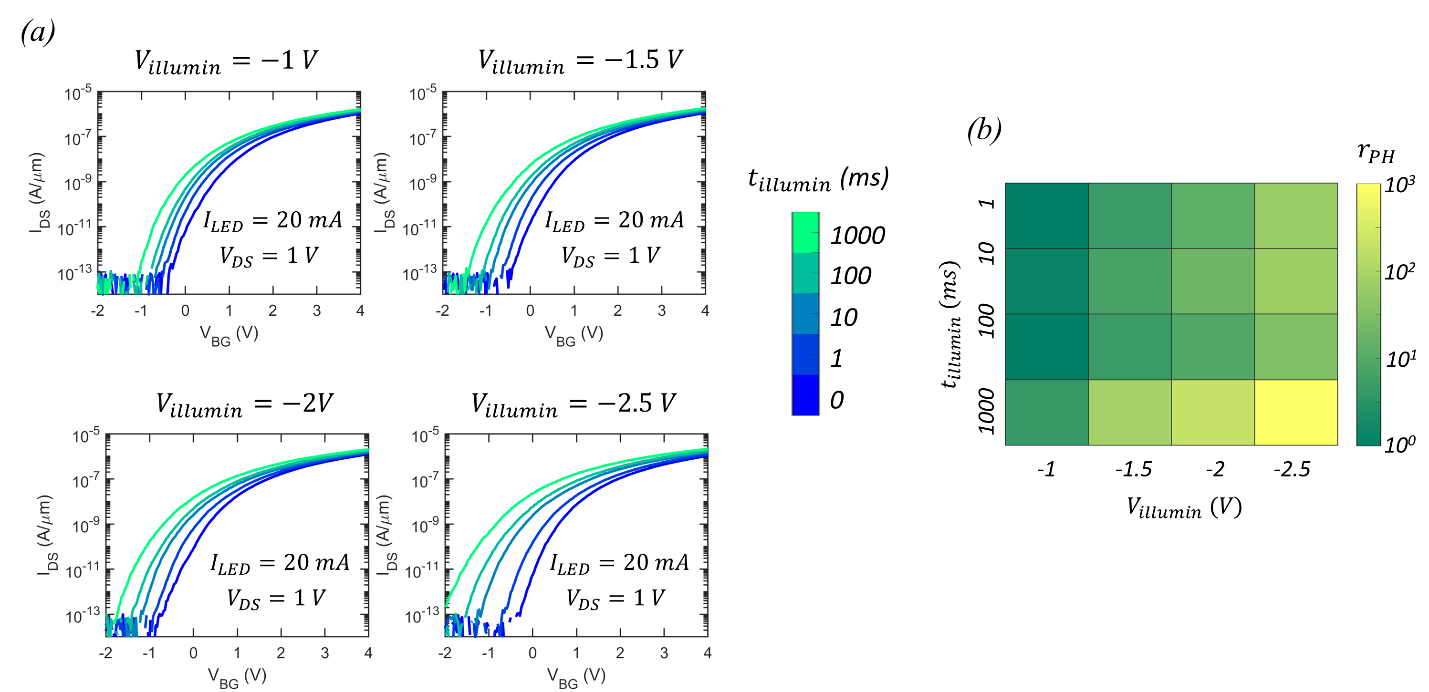


***Supplementary Figure 12***


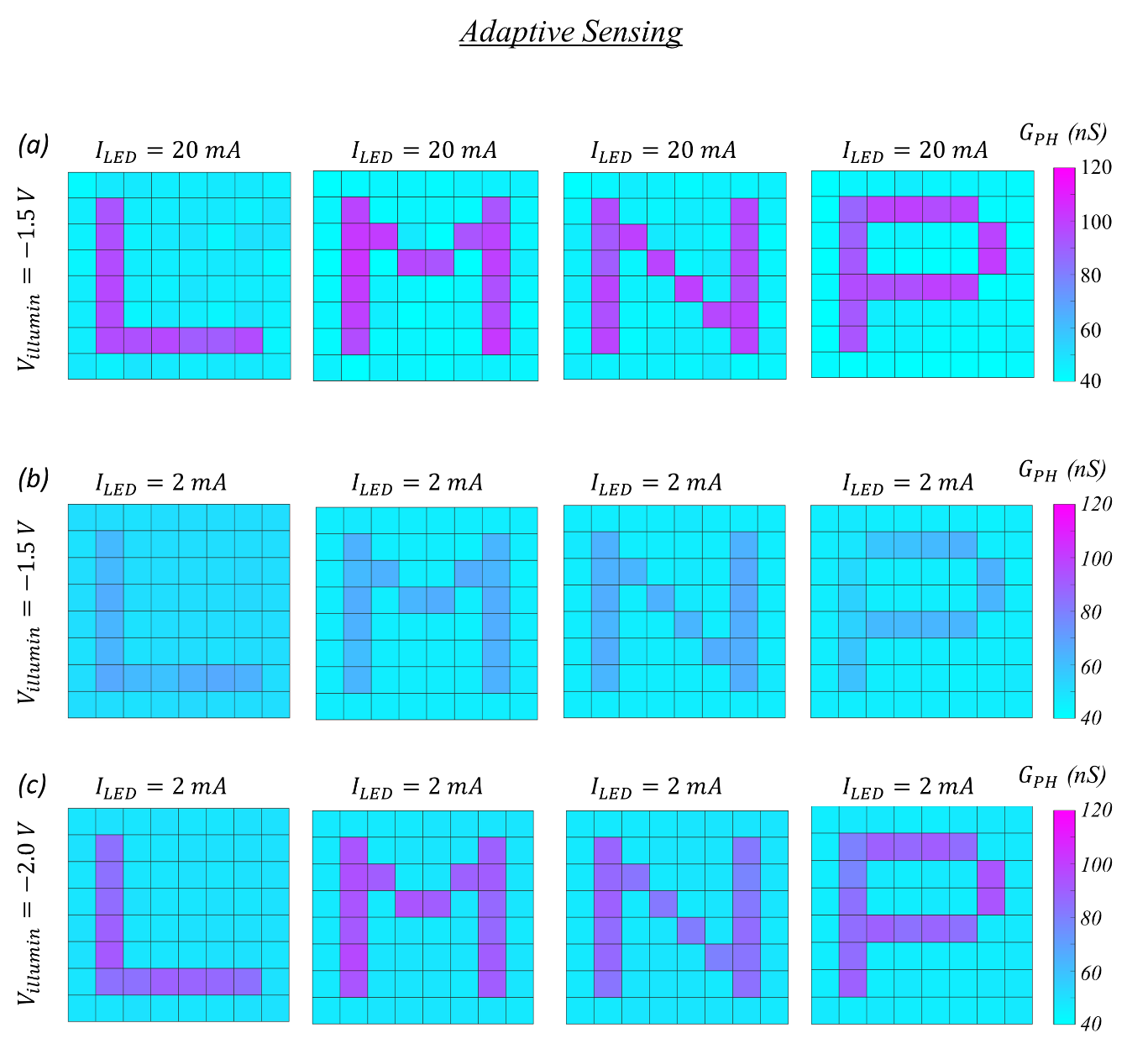


***Supplementary Figure 12****. a) Colormap of persistent photoconductivity (*$G_{\mathrm{PH}}$*) measured at* $V_{\mathrm{BG}}$ *= 0 V, when the* $T_{\mathrm{PT}}$ *corresponding to the 8 × 8 array of the crypto engines are exposed to 8 × 8 pixelated images of the letters, ‘L’, ‘M’, ‘N’, and ‘P’, obtained through the LED illumination for* $t_{\mathrm{illumin}}$ *= 100 ms with* $V_{\mathrm{illumin}}$ *= -1.5 V. The bright pixels correspond to* $I_{\mathrm{LED}}$ *= 20 mA. Clearly, MoS_2_ memtransistors integrated with the crypto engines are able to accurately transcribe the optical information into electrical response. Transcription of 8 × 8 pixelated images of the letters, ‘L’, ‘M’, ‘N’, and ‘P’ for dimmer LED illuminations (*$I_{\mathrm{LED}}$ *= 2 mA) using b)* $V_{\mathrm{illumin}}$ *= -1.5 V and c)* $V_{\mathrm{illumin}}$ *= -2.0 V. This demonstration highlights the advantages of gate-tunability of persistent photoconductivity in achieving adaptation to the illumination levels similar to the rod and cone neurons found in the visual system of primates.*

***Supplementary Figure 13***

***Supplementary Figure 13.*** *Transfer characteristics of 64 monolayer MoS_2_ memtransistors across the 8 × 8 array of the crypto engines measured in dark (pink) and post illumination (yellow). b) Compiled device-to-device variation in photoresponse. c) Colormap of distribution of ratio of post-illumination photoconductance to dark conductance (*$r_{\mathrm{PH}}$*) measured at* $V_{\mathrm{BG}}$ *= 0 V. The mean and standard deviation values for* $MR$ *were found to be 1.6 ×10^4^ and standard deviations of 3.6×10^4^, respectively.*


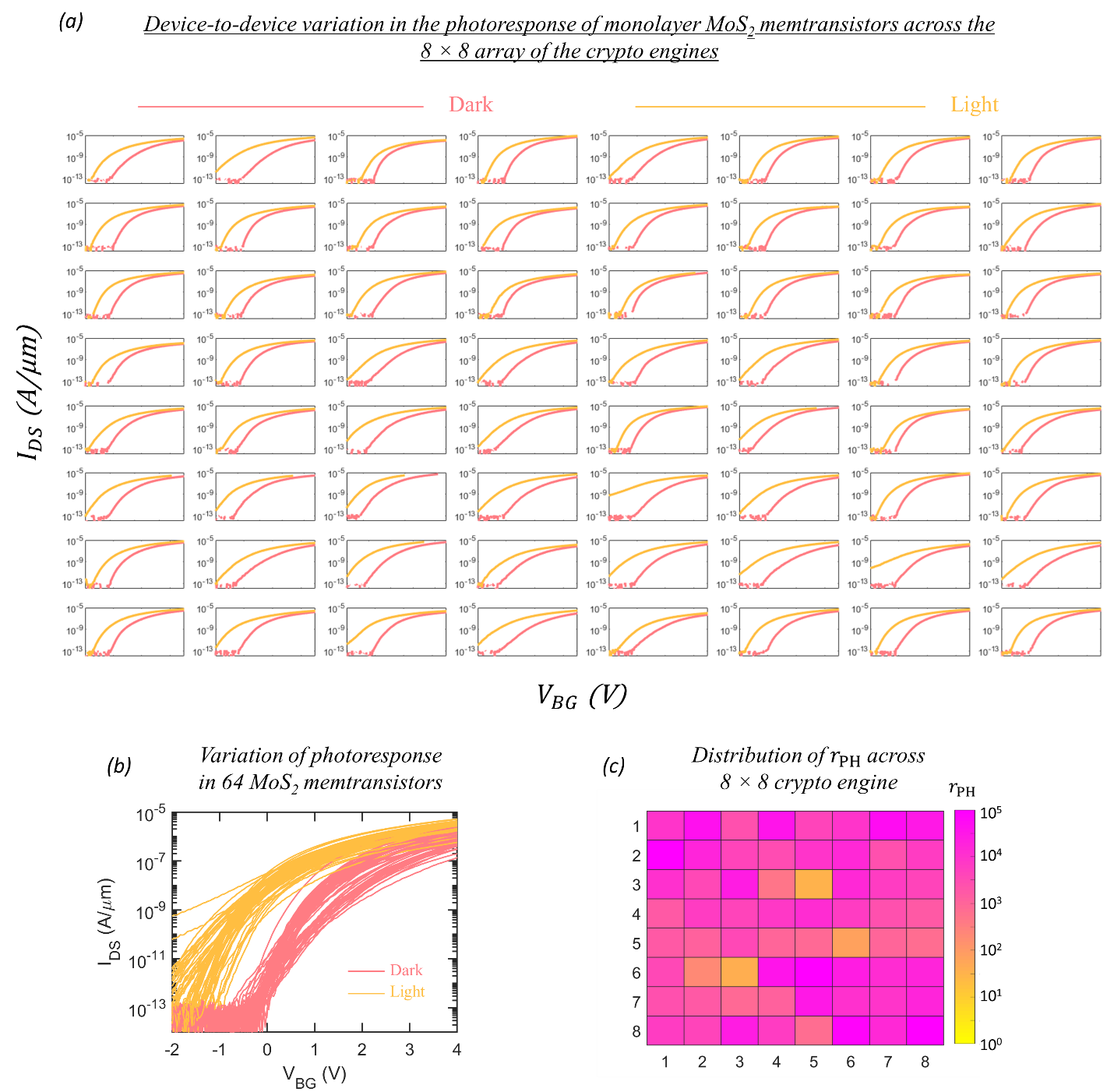


***Supplementary Figure 14***

***Supplementary Figure 14.*** *The input voltage waveforms applied to different nodes when a crypto engine is selected for encoding.* $V_{N1}$*,* $V_{N2}$*,* $V_{N6}$*, and* $V_{N7}$ *are held constant at 1 V, 5 V, 0 V and 0 V, respectively, throughout the encryption process.* $V_{N8}$ *and* $V_{N9}$ *are enabled only during the encoding cycle, with 1 V and 5 V applied to the respective nodes. Finally,* $V_{N4}$ *cycles between* $V_{\mathrm{illumin}}$ *= -4 V,* $V_{\mathrm{encoding}}$ *= 0 V, and* $V_{\mathrm{erase}}$ *= 11 V, during photosensing, encoding, and reset cycles, respectively.*


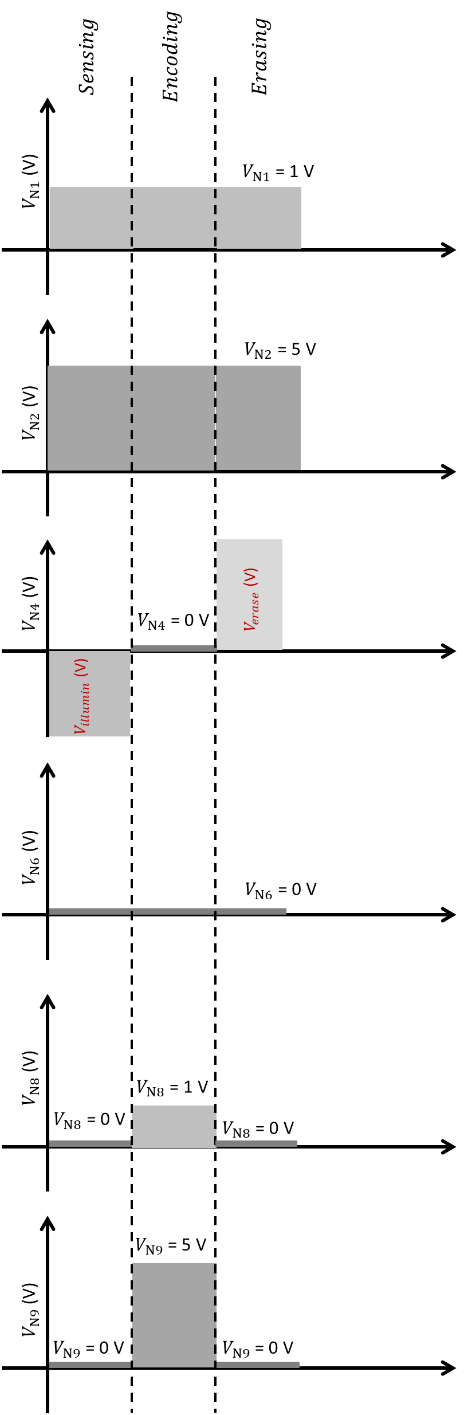


***Supplementary Figure 15***

***Supplementary Figure 15.*** *Colormap of the distribution of the* $G_{\mathrm{WGNA}}$ *for different noise standard deviations (* $\sigma_{G}$*).*


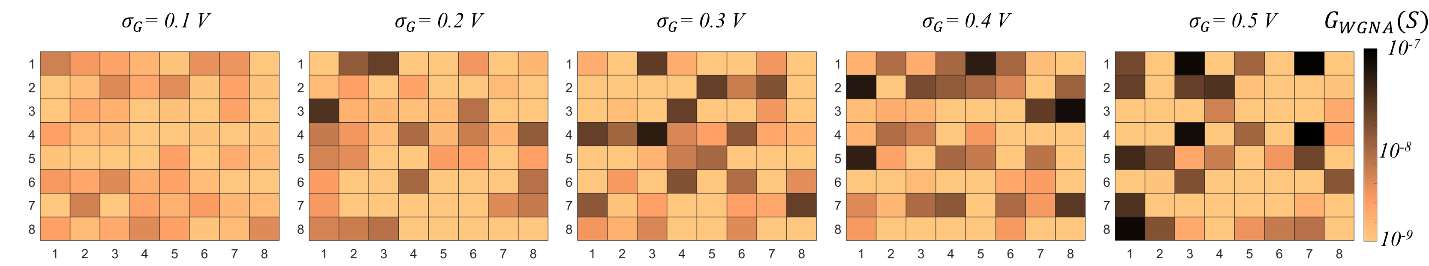


***Supplementary Figure 16***

***Supplementary Figure 16.*** *Transfer characteristics of a representative* $T_{\mathrm{SN}}$*, when measured at a drain bias of* $V_{\mathrm{DS}} =1$*V in both logarithmic and linear scale.*


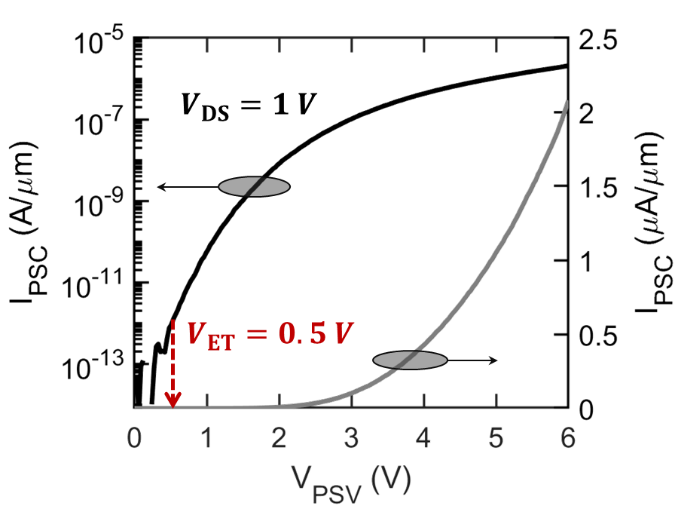


***Supplementary Figure 17***

***Supplementary Figure 17.*** *Number of brute force trials (BFTs) required to decode the letter ‘N’ for different image size (S) as a function of* $\sigma_{G}$*.*


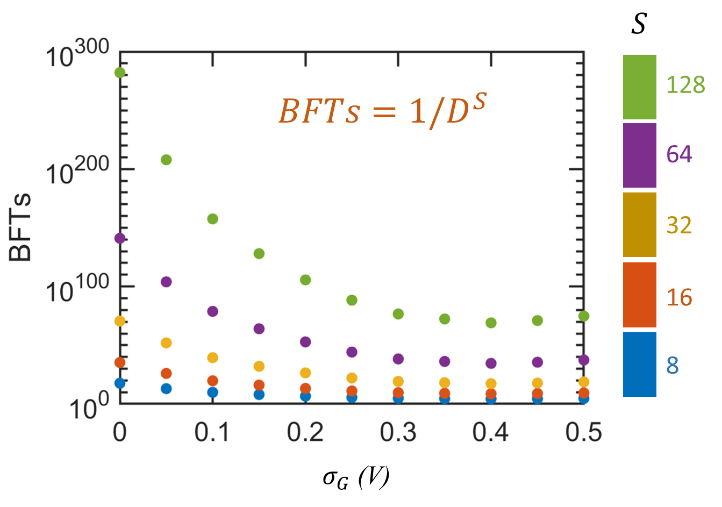


***Supplementary Note 1***

A detailed quantitative comparison of energy consumption is difficult owing to the diverse nature of the applications and experimental setups, however, a qualitative assessment can be presented. There has been several reports on lightweight cryptographic primitives that offer adequate security measures for resource-constrained devices. This includes block and stream ciphers as well as hash functions. For example, Bahrami *et. al* proposed a lightweight stream algorithm for the encryption of gray-scale images.^1^ While the proposed scheme can be implementated on a 32-bit microcontroller, ^2^ hardware demonstration is yet to be realized. pure software implementation and is not suitable for hardware applications. Symmetric encryption is the most popular design choice for image encryption. The advanced encryption standard (AES) is a classic example of symmetric encryption. AES is an energy-efficient encryptor and a widely employed security system.^3^ However the AES algorithm is computationally expensive and is hard to implement at the sensor node to manage real-time applications. Some of the other lightweight schemes include CLEFIA^4^, PRESENT^5^, PRINCE^6^, and SIMON and SPECK^7^, which can be implemented with a very small area and power using serial data paths. However, these serial architectures lag in performance and do not essentially provide energy optimal operation. Moreover, serial architectures are highly susceptible to side-channel attacks.^8^ In addition to the attacks, the amount of energy consumed in the above-mentioned lightweight cryptographic primitives ranges from ~ 10 nanojoules to ~ 100 microjoules.^9^ Recently, Pham *et. al* have proposed an architecture to construct visual sensor motes employing FPGA (field-programmable gate array) platform. This architecture is predicted to be 20 times more efficient than other common visual sensor motes but consumes ~2 mJ of energy.^10^ Similarly, Aziz *et. al* proposed a hardware architecture for efficient DWT (discrete wavelet transform) coding of sensed images selectively using the JPEG2000 codec. This architecture consumes ~34 mW of power in which ~3 mW of power is consumed in DWT for image processing and compression. Sensor motes with 16-bit processor may perform efficient sensing and transmission functions, but specialized modules for image processing are required for higher efficiency, which leads to energy and area overhead.^11-13^ The key contribution of our work is developing a crypto engine that integrates sensing, storage, compute, and security at miniscule energy expebnditure of few hundreds of picojoules.

***Supplementary Figure 18***

***Supplementary Figure 18.*** *The training and inference accuracy as a function of the number of hidden layers (* $N_{\mathrm{Layer}}$*) and number of epochs for a deep neural network for MNIST digit classification. Training accuracy of 100% and testing accuracy of > 98% was achieved beyond 50 epochs irrespective of* $N_{\mathrm{Layer}}$*.*


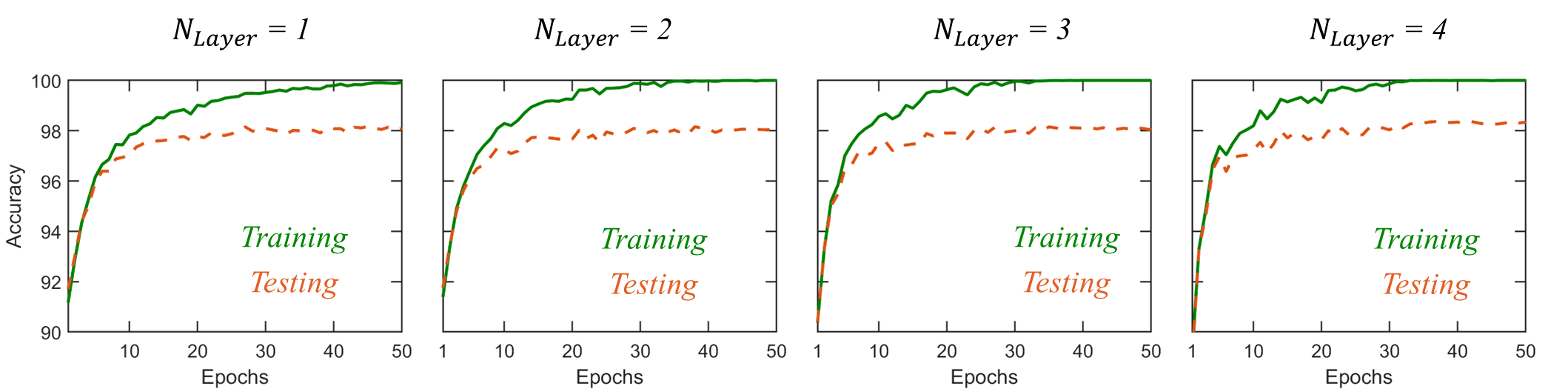


***Supplementary Figure 19***


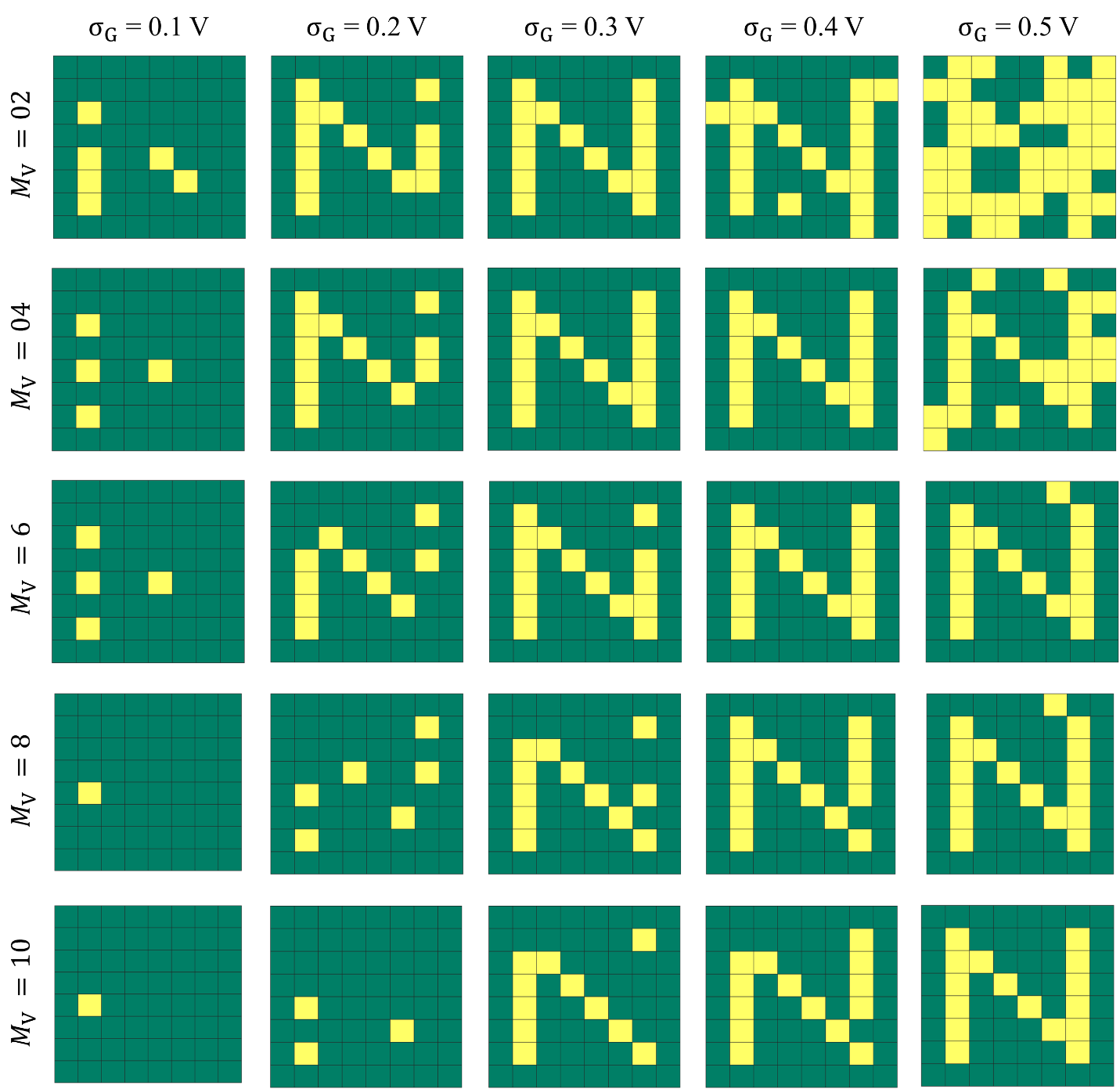


***Supplementary Figure 19.*** *Decoding of the images of the letter ‘N’, for different* $\sigma_{G}$ *and for different number of mandated votes (*$M_{V}$*) required to mark a pixel as bright for P = 50.*

***Supplementary Figure 20***

***Supplementary Figure 20.*** *Colormap of CC between the original and the decrypted image of the letter ‘N’ as a function of* $\sigma_{G}$ *and* $M_{V}$*, when encryption is done by different size of encoding population (*$P$*) with encoding threshold of* $V_{\mathrm{ET}}$ *= 0.5 V. As expected, the optimum number of* $M_{V}$*for accurate decryption is found to be different for similar* $\sigma_{G}$*. Therefore, without the prior knowledge of the* $\sigma_{G}$*and* $P$*,used by the encoder it is difficult to decode the information.*


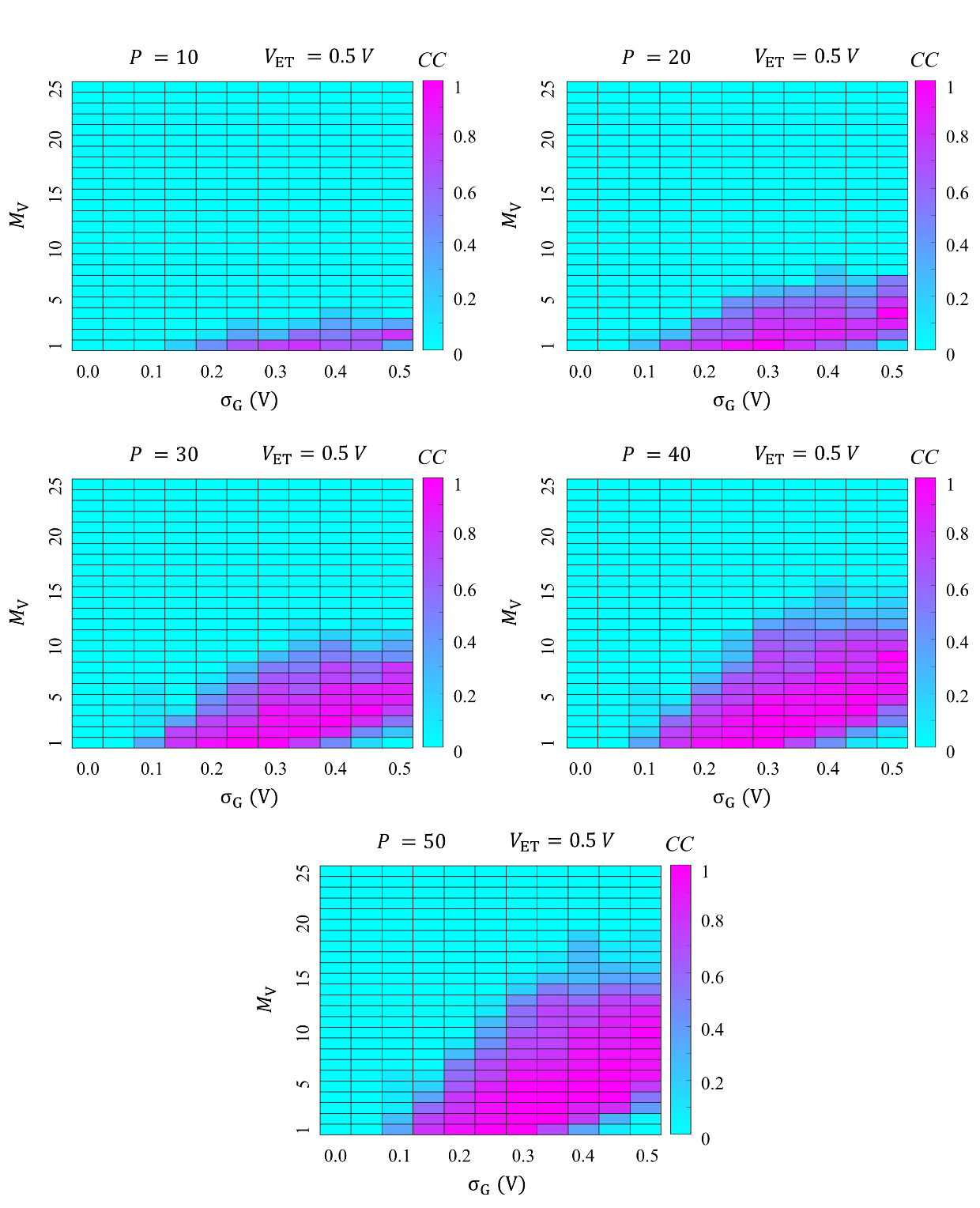


***Supplementary Figure 21***

***Supplementary Figure 21.*** *The encryption of the letter ‘N’, by the encoders with different* $V_{\mathrm{ET}}$*, at different* $\sigma_{G}$*. If* $V_{\mathrm{PSV}}$*>* $V_{\mathrm{ET}}$*, the encryption process or the communication is insecure. For* $V_{\mathrm{ET}}$ *values slightly greater than* $V_{\mathrm{PSV}}$*, there are more threshold crossing events even for low* $\sigma_{G}$*, whereas, for* $V_{\mathrm{ET}}$ *values further from* $V_{\mathrm{PSV}}$*, there are limited threshold crossing events even for high* $\sigma_{G}$*.*


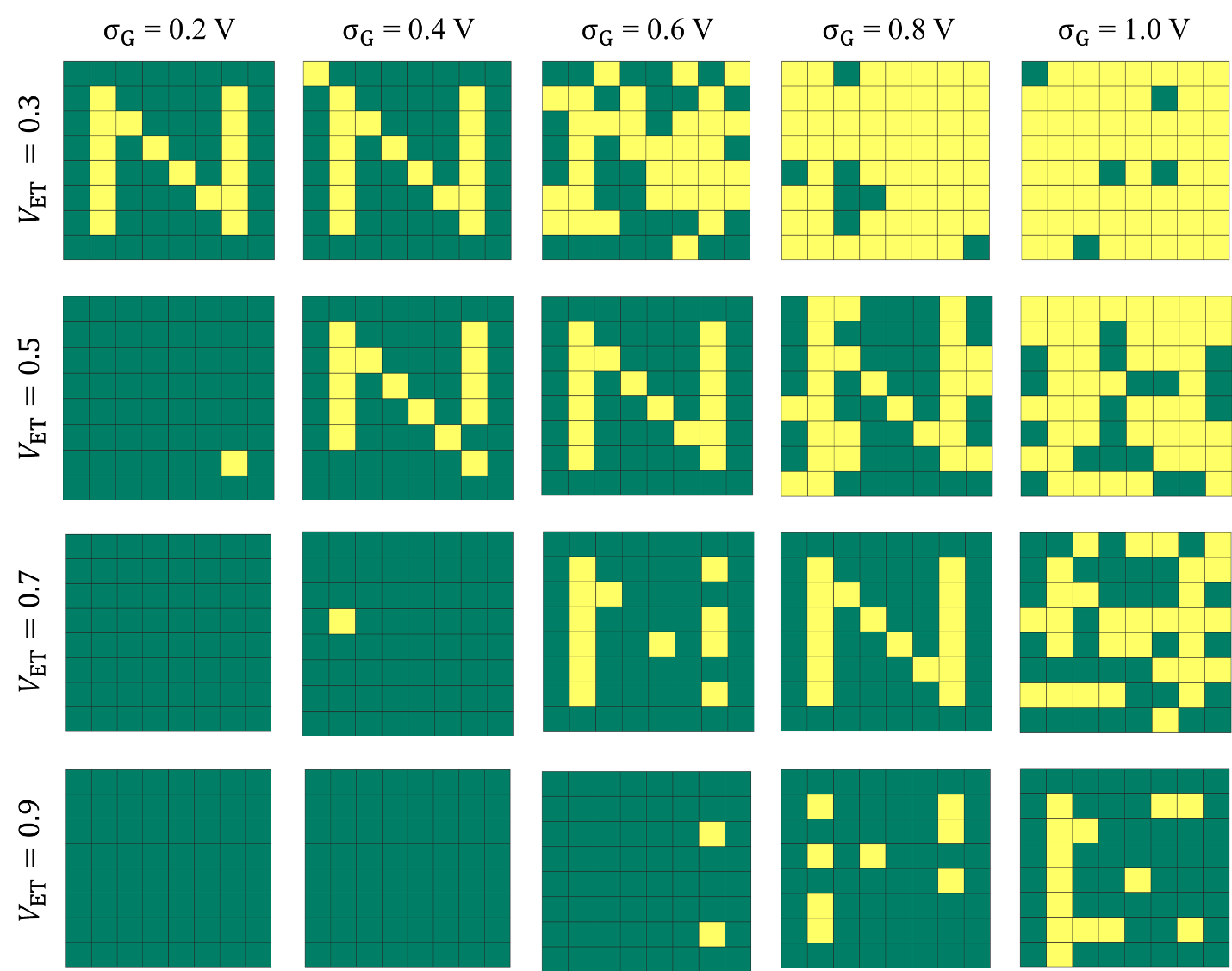


***Supplementary Figure 22***

***Supplementary Figure 22.*** *The colormap of CC between the original and the decrypted image of the letter ‘N’ as a function of* $V_{\mathrm{ET}}$ *of the encoder and* $M_{V}$ *mandated by the decoder for various* $\sigma_{G}$*at a given population size of P=50.*


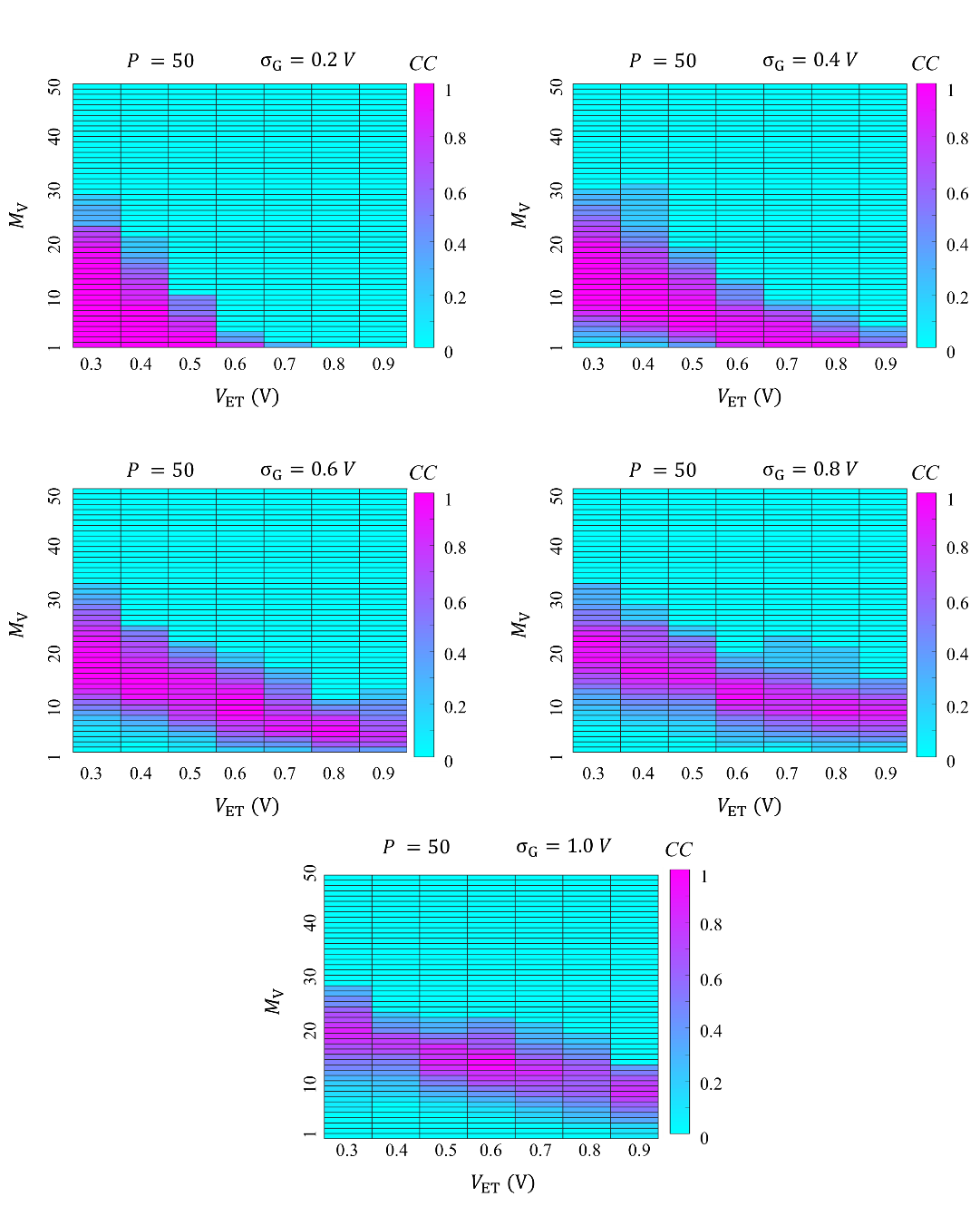


References

1 Bahrami, S. & Naderi, M. Image encryption using a lightweight stream encryption algorithm. *Advances in Multimedia* **2012** (2012).

2 Janakiraman, S., Thenmozhi, K., Rayappan, J. B. B. & Amirtharajan, R. Lightweight chaotic image encryption algorithm for real-time embedded system: Implementation and analysis on 32-bit microcontroller. *Microprocessors and Microsystems* **56**, 1-12 (2018).

3 Gonçalves, D. D. O. & Costa, D. G. A survey of image security in wireless sensor networks. *Journal of Imaging* **1**, 4-30 (2015).

4 Proença, P. & Chaves, R. in *2011 21st International Conference on Field Programmable Logic and Applications.* 512-517 (IEEE).

5 Bogdanov, A. *et al.* in *International workshop on cryptographic hardware and embedded systems.* 450-466 (Springer).

6 Borghoff, J. *et al.* in *International conference on the theory and application of cryptology and information security.* 208-225 (Springer).

7 Beaulieu, R. *et al.* in *Proceedings of the 52nd Annual Design Automation Conference.* 1-6.

8 Bogdanov, A. in *International Workshop on Selected Areas in Cryptography.* 84-95 (Springer).

9 Alioto, M. Trends in hardware security: From basics to ASICs. *IEEE Solid-State Circuits Magazine* **11**, 56-74 (2019).

10 Pham, D. M. & Aziz, S. M. Object extraction scheme and protocol for energy efficient image communication over wireless sensor networks. *Computer Networks* **57**, 2949-2960 (2013).

11 Pham, D. M. & Aziz, S. M. in *2013 IEEE Eighth International Conference on Intelligent Sensors, Sensor Networks and Information Processing.* 260-264 (IEEE).

12 Hasan, K. K., Ngah, U. K. & Salleh, M. F. M. Efficient hardware-based image compression schemes for wireless sensor networks: A survey. *Wireless personal communications* **77**, 1415-1436 (2014).

13 Pham, D. M. & Aziz, S. M. in *2011 IFIP 9th International Conference on Embedded and Ubiquitous Computing.* 100-105 (IEEE).

14 Lopez-Sanchez, O., Lembke, D., Kayci, M., Radenovic, A. & Kis, A. Ultrasensitive photodetectors based on monolayer MoS2. *Nat Nanotechnol* **8**, 497-501, doi:10.1038/nnano.2013.100 (2013).

15 Ge, R. *et al.* Atomristor: Nonvolatile Resistance Switching in Atomic Sheets of Transition Metal Dichalcogenides. *Nano Lett* **18**, 434-441, doi:10.1021/acs.nanolett.7b04342 (2018).

16 Liu, C. *et al.* A semi-floating gate memory based on van der Waals heterostructures for quasi-non-volatile applications. *Nat Nanotechnol* **13**, 404-410, doi:10.1038/s41565-018-0102-6 (2018).

17 Wang, M. *et al.* Robust memristors based on layered two-dimensional materials. *Nat Electron* **1**, 130-136, doi:10.1038/s41928-018-0021-4 (2018).

18 Bertolazzi, S., Krasnozhon, D. & Kis, A. Nonvolatile memory cells based on MoS2/graphene heterostructures. *ACS Nano* **7**, 3246-3252, doi:10.1021/nn3059136 (2013).

19 Choi, M. S. *et al.* Controlled charge trapping by molybdenum disulphide and graphene in ultrathin heterostructured memory devices. *Nat Commun* **4**, 1624, doi:10.1038/ncomms2652 (2013).

20 Vu, Q. A. *et al.* Two-terminal floating-gate memory with van der Waals heterostructures for ultrahigh on/off ratio. *Nat Commun* **7**, 12725, doi:10.1038/ncomms12725 (2016).

21 Roy, K. *et al.* Graphene-MoS2 hybrid structures for multifunctional photoresponsive memory devices. *Nat Nanotechnol* **8**, 826-830, doi:10.1038/nnano.2013.206 (2013).

22 Seo, S. *et al.* Artificial optic-neural synapse for colored and color-mixed pattern recognition. *Nat Commun* **9**, 5106, doi:10.1038/s41467-018-07572-5 (2018).

23 Wang, S. *et al.* A MoS2 /PTCDA Hybrid Heterojunction Synapse with Efficient Photoelectric Dual Modulation and Versatility. *Adv Mater* **31**, e1806227, doi:10.1002/adma.201806227 (2019).

24 Lee, D. *et al.* Multibit MoS2 Photoelectronic Memory with Ultrahigh Sensitivity. *Adv Mater* **28**, 9196-9202, doi:10.1002/adma.201603571 (2016).

25 Xiang, D. *et al.* Two-dimensional multibit optoelectronic memory with broadband spectrum distinction. *Nat Commun* **9**, 2966, doi:10.1038/s41467-018-05397-w (2018).

26 Wang, Q. *et al.* Nonvolatile infrared memory in MoS2/PbS van der Waals heterostructures. *Sci Adv* **4**, eaap7916, doi:10.1126/sciadv.aap7916 (2018).

27 Tian, H. *et al.* A Dynamically Reconfigurable Ambipolar Black Phosphorus Memory Device. *ACS Nano* **10**, 10428-10435, doi:10.1021/acsnano.6b06293 (2016).

28 Li, D. *et al.* Nonvolatile Floating-Gate Memories Based on Stacked Black Phosphorus-Boron Nitride-MoS2Heterostructures. *Advanced Functional Materials* **25**, 7360-7365, doi:10.1002/adfm.201503645 (2015).

29 Lee, Y. T. *et al.* Nonvolatile Charge Injection Memory Based on Black Phosphorous 2D Nanosheets for Charge Trapping and Active Channel Layers. *Advanced Functional Materials* **26**, 5701-5707, doi:10.1002/adfm.201602113 (2016).

30 Wang, J. *et al.* Floating gate memory-based monolayer MoS2 transistor with metal nanocrystals embedded in the gate dielectrics. *Small* **11**, 208-213, doi:10.1002/smll.201401872 (2015).

31 Dodda, A. *et al.* Stochastic resonance in MoS2 photodetector. *Nat Commun* **11**, 4406, doi:10.1038/s41467-020-18195-0 (2020).

32 Jayachandran, D. *et al.* A low-power biomimetic collision detector based on an in-memory molybdenum disulfide photodetector. *Nat Electron* **3**, 646-655, doi:10.1038/s41928-020-00466-9 (2020).

33 Lipatov, A., Sharma, P., Gruverman, A. & Sinitskii, A. Optoelectrical Molybdenum Disulfide (MoS2)--Ferroelectric Memories. *ACS Nano* **9**, 8089-8098, doi:10.1021/acsnano.5b02078 (2015).

34 Mennel, L. *et al.* Ultrafast machine vision with 2D material neural network image sensors. *Nature* **579**, 62-66, doi:10.1038/s41586-020-2038-x (2020).

35 Bessonov, A. A. *et al.* Layered memristive and memcapacitive switches for printable electronics. *Nat Mater* **14**, 199-204, doi:10.1038/nmat4135 (2015).

36 Shao, B. *et al.* Crypto primitive of MOCVD MoS2 transistors for highly secured physical unclonable functions. *Nano Research*, doi:10.1007/s12274-020-3033-0 (2020).

37 Wang, Z. *et al.* Memristors with diffusive dynamics as synaptic emulators for neuromorphic computing. *Nat Mater* **16**, 101-108, doi:10.1038/nmat4756 (2017).

38 Yao, P. *et al.* Face classification using electronic synapses. *Nat Commun* **8**, 15199, doi:10.1038/ncomms15199 (2017).

39 Wu, S. *et al.* Bipolar resistance switching in transparent ITO/LaAlO(3)/SrTiO(3) memristors. *ACS Appl Mater Interfaces* **6**, 8575-8579, doi:10.1021/am501387w (2014).

40 Ohno, T. *et al.* Short-term plasticity and long-term potentiation mimicked in single inorganic synapses. *Nat Mater* **10**, 591-595, doi:10.1038/nmat3054 (2011).

41 Ionescu, O., Besleaga, C., Dumitru, V. & Pricop, E. in *2020 12th International Conference on Electronics, Computers and Artificial Intelligence (ECAI).* 1-4 (IEEE).

42 Nili, H. *et al.* Hardware-intrinsic security primitives enabled by analogue state and nonlinear conductance variations in integrated memristors. *Nat Electron* **1**, 197-202, doi:10.1038/s41928-018-0039-7 (2018).

43 Zhang, R. *et al.* Nanoscale diffusive memristor crossbars as physical unclonable functions. *Nanoscale* **10**, 2721-2726, doi:10.1039/c7nr06561b (2018).

44 Jiang, H. *et al.* A novel true random number generator based on a stochastic diffusive memristor. *Nat Commun* **8**, 882, doi:10.1038/s41467-017-00869-x (2017).

45 Ge, N. *et al.* An efficient analog Hamming distance comparator realized with a unipolar memristor array: a showcase of physical computing. *Sci Rep* **7**, 40135, doi:10.1038/srep40135 (2017).

46 Feldmann, J., Youngblood, N., Wright, C. D., Bhaskaran, H. & Pernice, W. H. All-optical spiking neurosynaptic networks with self-learning capabilities. *Nature* **569**, 208-214 (2019).

47 Yin, L. *et al.* Synaptic silicon-nanocrystal phototransistors for neuromorphic computing. *Nano Energy* **63**, 103859 (2019).
